# Spontaneous Tubercular Enterocutaneous Fistula Developing in the Scar of a Surgery Done 14 Years Earlier

**DOI:** 10.4103/1319-3767.56107

**Published:** 2009-10

**Authors:** Onkar Singh, Shilpi Gupta, Sonia Moses, Devendra K. Jain

**Affiliations:** Department of Surgery, MGM Medical College and MY Hospital, Indore, India

**Keywords:** Complications of abdominal tuberculosis, spontaneous tubercular enterocutaneous fistula, spontaneous enterocutaneous fistula, tubercular enteritis

## Abstract

We report a case of spontaneous tubercular enterocutaneous fistula, which occurred after a long interval of 14 years after an appendicectomy. A 32-year-old male presented with the complaint of fecal matter coming out continuously from an opening present over the scar of previous surgery. The only significant past history was that of appendicectomy done 14 years back for acute appendicitis (nontubercular). Histopathology of tissue taken from the margins of the fistulous opening showed caseating granuloma, consistent with tuberculosis. Treatment was provided successfully in the form of fistulectomy and right hemicolectomy with ileotransverse anastomosis along with a 9-month course of four-drug antitubercular treatment. Regular follow-up for the last 2 years has been uneventful.

Nowadays, with the use of antituberculous drugs, the incidence of abdominal tuberculosis (TB) and its complications has decreased. As a result, general surgeons only rarely come across tubercular enterocutaneous fistulae. Spontaneous occurrence of such fistulae is even rarer. Young surgeons therefore may not be aware about the management of such cases. By reporting this case we wish to demonstrate the possibility of spontaneous development of an enterocutaneous fistula over a surgical scar after a very long interval (in this case, 14 years after an appendicectomy) and to show that primary tubercular enteritis could be a cause, especially in long standing cases.

## CASE REPORT

A 30-year-old male of asthenic build presented with the complaint that fecal matter was coming out through an opening in the right iliac fossa region of his abdomen for the past 6 months. Initially, he had developed a swelling in the skin of the right iliac fossa over the scar of an appendectomy done 14 years back. The swelling had ruptured after 7-8 days, to discharge fecal matter mixed with pus. Within a week, the opening healed, but the swelling reappeared 7-8 days later, only to rupture again after a few days. This cycle of swelling followed by rupture was associated with evening rise of temperature. The fever was of moderate degree and was associated with the swelling stage only. His appetite was adequate and he was passing stools and urine normally.

**Figure 1:**
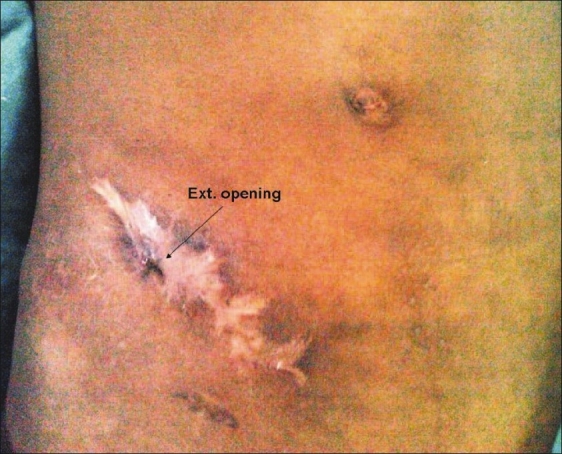
Fistulous opening over the scar of a previous appendicectomy

On examination, the patient was moderately nourished and there were no features of anemia, hypoproteinemia, dehydration, or electrolyte disturbances. A 0.5 cm × 0.5 cm fistulous opening with fecal soiling was seen over the scar of a previous surgery in the right iliac fossa [Figure 1]. No mass could be palpated in the abdomen. The patient had no history of any other surgery or hospitalization. There was no history suggestive of any chronic disease such as TB, diabetes mellitus, or ischemic heart disease. Biopsy of the inflamed appendix and of an enlarged lymph node, taken at the time of the surgery done 14 years back, had not shown caseating granuloma but only nonspecific inflammatory reaction.

**Figure 2:**
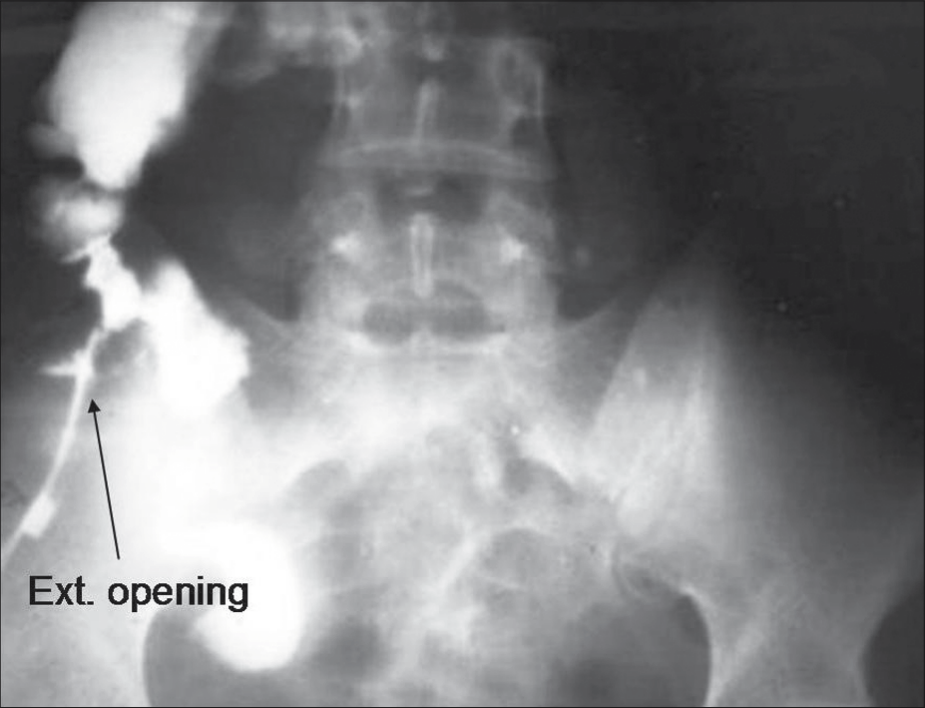
Fistulogram demonstrating narrowing involving the ileocaecal junction and the terminal ileum, and a fistulous communication between the terminal ileum and the skin

We took X-rays of the chest and abdomen, both of which were normal. Abdominal sonography revealed bowel-wall thickening in the ileocecal region and enlarged mesenteric lymph nodes, as well as a fistulous tract between the terminal ileum and the skin. A percutaneous fistulogram was obtained, which revealed a narrowing involving the ileocaecal junction and the terminal ileum, and a fistulous tract connecting the terminal ileum to the opening in the skin in the right iliac fossa [Figure 2]. Colonoscopy was performed and showed a normal colon. Tissue specimen taken from the margins of the fistulous opening on the skin was subjected to Ziehl-Nielsen staining for acid-fast bacilli (AFB) but was negative. However, histopathological examination of tissue specimens from the same site showed caseating granuloma, consistent with tuberculosis. The tuberculin test was weakly positive.

Four-drug antitubercular therapy was started. The patient was sent home with the advice to return for follow-up after 6 weeks. At 6 weeks, the fistula did not show any signs of healing. Although it was a low-output fistula (with an output of about 100 ml/day), in view of the long history and the fact that the edges of fistula had completely epithelized, exploration was planned after complete bowel preparation.

Intraoperatively, the ileocecal portion was found to be clumped and the site of fistula was seen to be the terminal ileum. The terminal ileum was dilated and its walls and the mesentery were thickened. Fistulectomy with right hemicolectomy was performed along with ileotransverse anastomosis. Histopathologically, the fistulous tract showed transmural dense chronic inflammatory cellular infiltration and histiocytic proliferation with Langhans-type giant cell reaction. Histological examination of the resected specimens revealed ‘casseous granuloma with central necrosis’ in the ileocecal portion and mesenteric lymph nodes, which is typical of tuberculous involvement. TB of the lungs or of any other organ was not demonstrated. The patient's postoperative course was uneventful and four- drug antitubercular therapy was continued for a further 8 months. Follow-up over 2 years has been uneventful and antitubercular therapy has been stopped.

## DISCUSSION

Intestinal TB continues to be a common problem in many developing countries.[1] The segment of bowel that is most frequently involved by abdominal TB is the ileocecal region, possibly because of the greater physiological stasis, greater rate of fluid and electrolyte absorption, minimal digestive activity, and the abundance of lymphoid tissue at this site.[2] The various complications of intestinal TB include bowel obstruction (31.7%), intestinal perforation (4.9%), enterocutaneous fistula formation (2.4%), and small bowel volvulus due to mesenteric lymphadenitis (2.4%).[1] Obstruction is a well known common complication of tuberculous enteritis. Perforation is an uncommon complication, and tubercular enterocutaneous fistula is very rare.[3,4] Spontaneous tubercular enterocutaneous fistulae, in the strict sense, are not spontaneous, as their development is secondary to underlying intestinal diseases. Such conditions include Crohn disease,[5] malignancy,[6] infectious processes such as tuberculosis[7] and typhoid fever,[8] radiation exposure,[9] diverticulitis, vascular insufficiency, and mesenteric ischemia.[10] Other causes include diverticulosis, appendicitis, pancreatitis[4] and, in rare instances, congenital hernias[11] and even Littré hernia.[12] Fistulae that occur after some primary intervention (surgery or radiotherapy) are also considered as ‘spontaneous’ if they develop 30 days after the primary intervention.[13]

Preoperative diagnosis of underlying pathology as abdominal TB is often difficult[14] for several reasons. The absence of radiological evidence of pulmonary TB results in a low index of suspicion. Also, the condition may clinically mimic other diseases such as Crohn disease, neoplasm, and appendicular mass.[15,16] Histopathological examination of tissue specimens taken from the margins of the fistula may show the specific finding of caseous granulomas with central necrosis, as in our case. Accurate diagnosis of the fistula can be made by doing a fistulogram, using water-soluble iodinated media. Combination with barium studies can accurately establish the site of the fistula in case of difficulty. CECT can show the actual tract of the fistula and also locate any associated pathology.[9,17]

Measurement of the output of the fistula and classification into low- and high-output varieties has implications for the management, as high-output fistulae usually require surgical intervention, while low-output fistulae in well- preserved individuals may be managed conservatively.[11] Drainage of associated intra-abdominal abscesses, along with appropriate antibiotic therapy, is also essential.[9] While some fistulae close spontaneously with medical management alone, many require surgical intervention.[10] As is the case with perforation in intestinal TB, tubercular enterocutaneous fistula can also be treated with fistulectomy and resection of the involved segment of intestine followed by an end-to-end anastomosis. Simple fistulectomy and closure of the intestinal end of fistula may lead to leak or recurrence of fistula.[18] In critically ill patients, exteriorization of both ends (ostomy and mucus fistula) should be considered temporarily. In our case, the patient was not nutritionally depleted and thus never required hyperalimentation, which is usually needed for management of such cases.

Although, primary abdominal TB is considered a common condition, spontaneous enterocutaneous fistula in such cases is rare. To the best of our knowledge, this is the first report of a tubercular enterocutaneous fistula developing spontaneously in the scar of appendicectomy done many years (14 years, in this case) earlier. The possibility of TB being the underlying cause should be kept in mind in any case of long-standing fistula in a surgical scar.

## References

[1] Alper Akino Glu, Ilter Bilgin (1988). Tuberculous Enteritis and Peritonitis. Can J Surg.

[2] Paustian FF, Bockus HL (1959). So-called primary ulcerohypertrophic ileocecal tuberculosis. Am J Med.

[3] Rao PL, Mitra SK, Pathak IC (1979). Spontaneous tuberculous enteroumbilical fistulas. Am J Gastroentrol.

[4] Ceccherini E, Sereni P, Felicioni L, Testi W, Mancini S (1989). Tubercular enteral fistulas. Minerva Chir.

[5] Hollington P, Mawdsley J, Lim W, Gabe SM, Forbes A, Windsor AJ (2004). A 11 yr experience of enterocutaneous fistula. Br J Surg.

[6] Chamberlain RS, Kaufman HL, Danforth DN (1998). Enterocutaneous fistula in cancer patients: Etiology, management, outcome, and impact on further treatment. Am Surg.

[7] Pickhardt PJ, Bhalla S, Balfe DM (2002). Acquired Gastrointestinal Fistulas: Classification, Etiologies, and Imaging Evaluation. Radiology.

[8] Otaigbe BE, Anochie IC, Gbobo I (2006). Spontaneous Enterocutaneous Fistula; A Rare Presentation of Enteric Fever. J Natl Med Assoc.

[9] Chintamani, Badran R, Rk D, Singhal V, Bhatnagar D (2003). Spontaneous entrocutaneous Fistula 27-years Following Radiotherapy in a Patient of Carcinoma Penis. World J Surg Oncol.

[10] Stawicki SP, Braslow BM (2008). Gastrointestinal Fistulae. OPUS 12 Scientist.

[11] Leslie MD, Slater ND, Smallwood CJ (1983). Small bowel fistula from a Littre's hernia. Br J Surg.

[12] Ameh EA, Awotula OP, Amoah JN (2002). Spontaneous scrotal faecal fistula in infants. Pediatr Surg Int.

[13] Sitges-Serra A, Jaurrieta E, Sitges-Creus A (1989). Management of postoperative enterocutaneous fistulas: The role of parenteral nutrition and surgery. Br J Surg.

[14] Bhansali SU (1977). Abdominal tuberculosis: Experience with 300 cases. Am J Gastroenterol.

[15] Edington GM, Gilles HM (1976). Tuberculosis of the alimentary tract. Pathology in the Tropics.

[16] Walker-Smith J (1988). Abdominal tuberculosis. Disease of the Small Intestine in Childhood.

[17] Makanjuola D (1998). Is it Crohn's disease or intestinal T.B? CT analysis. Eur-J-Radiol.

[18] Ara C, Sogutlu G, Yildiz R, Kocak O, Isik B, Yilmaz S (2005). Spontaneous small bowel perforations due to intestinal tuberculosis should not be repaired by simple closure. J Gastrointest Surg.

